# Genome-Wide Analysis Characterization and Evolution of *SBP* Genes in *Fragaria vesca*, *Pyrus bretschneideri*, *Prunus persica* and *Prunus mume*

**DOI:** 10.3389/fgene.2018.00064

**Published:** 2018-03-02

**Authors:** Muhammad Abdullah, Yunpeng Cao, Xi Cheng, Awais Shakoor, Xueqiang Su, Junshan Gao, Yongping Cai

**Affiliations:** ^1^School of Life Sciences, Anhui Agricultural University, Hefei, China; ^2^School of Resources and Environment, Anhui Agricultural University, Hefei, China

**Keywords:** SBP-box, phylogenetic analysis, microsynteny, selection, functional divergence

## Abstract

The SQUAMOSA promoter binding protein (SBP)-box proteins are plant-specific transcriptional factors in plants. SBP TFs are known to play important functions in a diverse development process and also related in the process of evolutionary novelties. *SBP* gene family has been characterized in several plant species, but little is known about molecular evolution, functional divergence and comprehensive study of *SBP* gene family in Rosacea. We carried out genome-wide investigations and identified 14, 32, 17, and 17 *SBP* genes from four Rosacea species (*Fragaria vesca*, *Pyrus bretschneideri, Prunus persica* and *Prunus mume*, respectively). According to phylogenetic analysis arranged the SBP protein sequences in seven groups. Localization of SBP genes presented an uneven distribution on corresponding chromosomes of Rosacea species. Our analyses designated that the SBP genes duplication events (segmental and tandem) and divergence. In addition, due to highly conserved structure pattern of SBP genes, recommended that highly conserved region of microsyneteny in the Rosacea species. Type I and II functional divergence was detected among various amino acids in SBP proteins, while there was no positive selection according to substitutional model analysis using PMAL software. These results recommended that the purifying selection might be leading force during the evolution process and dominate conservation of SBP genes in Rosacea species according to environmental selection pressure analysis. Our results will provide basic understanding and foundation for future research insights on the evolution of the SBP genes in Rosacea.

## Introduction

Squamosa promoter binding proteins (SBP)-box genes family are present in all photosynthetic organisms from green algae to multicellular trees except animals, prokaryotes, and fungi. SBP-box genes encoded a family of plant-specific transcription factors (TFs), which are significant regulators of many biological processes, a particular transition phase, microsporogenesis, megasporogenesis, ripening of fruit, trichome development on sepals, stamen filament elongation and homeostasis.

The SBP domain consists of 79 amino acids with 10 conserved cysteine and histidine residues (Cardon et al., 1999; Zhang et al., 2015) that are involved in nuclear localization and DNA-binding. SBP proteins contain two zinc binding sites (Cys-Cys-Cys-His and Cys-Cys-His-Cys), among them, most have a three-stranded antiparallel beta-sheet (Yamasaki et al., 2004; Pan et al., 2017). The first SBP gene, *AmSBP1*, and *AmSBP2* were discovered from *Antirrhinum majus*, snapdragon, based on their specific interaction with a promoter sequence of SQUAMOSA identity gene (Klein et al., 1996; Cardon et al., 1999; Pan et al., 2017). The *SPL3* was the first SBP-box gene identified in *Arabidopsis*, which play a critical role in regulating flowering under long photoperiod (Cardon et al., 1997). Later on SBP gene family has been discovered and characterized several plants including *Oryza sativa* (Xie, 2006), *Silver Birch* (Lännenpää et al., 2004), *Solanum lycopersicum* (Salinas et al., 2012) Cotton (Zhang et al., 2014), green alga (Kropat et al., 2005), *Bryophyta* (Riese et al., 2007), and *Cucumis melon* (Ma et al., 2015). Previous studies reported that the SBP-box genes families of TFs have a potential role in the diverse development of plants. In *Arabidopsis* genome 16 SBP-box genes have been identified (Guo et al., 2008) and studied their role in the leaf development (Usami et al., 2009), early flowering (Gandikota et al., 2007), nutritional changes and reproductive stage (Jung et al., 2011), leaf primordia formation (Wang et al., 2008), gibberellic acid (GA) response (Zhang et al., 2007) and Copper homeostasis (Yamasaki et al., 2009). *SPL8* mutants express the difference in pollen sac development as compared to others and overexpression of SPL8 showed the effect on plant fertility through crosstalk signaling of GA. Similarly, *AtSPL11, AtSPL2*, and *AtSPL10* have a potential role in morphological changes in addition to reproductive phase and shoot maturation (Shikata et al., 2009). In rice, *OsSPL14* overexpression mutant altered the reproductive stage and significantly increased panicle branching and grain yield (Miura et al., 2010). In Chinese cabbage, the *BraSPL9-2* putative target of miR156 have been isolated and involved in the heading time (Wang et al., 2014). In Maize, SBP-box proteins have a critical role in the alteration of plant architecture and yield traits through the initiation of lateral primordia (Chuck et al., 2014). Genome-wide identification of different transcription factors like EIN3/EIL (Cao et al., 2017a), MYB (Cao et al., 2016b), WOX (Cao et al., 2017b) illuminates the post-transcriptional regulation in *Pyrus bretschneideri*. SBP-box genes performing the interphase role between phase change and homeostasis, due to plant diversification can be studied more deeply through SBP-box genes family. Interruption of specific SBP-box genes delays the juvenile phase, while articulate expression of others may lead to early flowering. The current investigation is searching for linking these SBP-box genes with identified flowering pathways. Hopefully, future studies will expose whether the homeostatic responses and transitions growth have the common themes in plants, whether SBP-box expresses like two sides of the same coin. Although SBP-box genes have been reported in several model plants, still this gene family has not been comprehensively studied in Rosacea. Herein, we conducted a comprehensive and systematic analysis of SBP-box genes from four Rosacea species, including Strawberry (*Fragaria vesca*) Chinese pear (*Pyrus bretschneideri*), Peach (*Prunus persica)*, and Mei (*Prunus mume*). Finally, we report SBP-box genes evolutionary relationship and divergence expression, gene duplication events based on the analysis of environmental selection pressure and microsynteny. In addition, the expression level of Strawberry SBP-box was examined in different tissue during the fruit development. Our results provided precise information of SBP-box genes that will be supportive for ongoing research on this important gene family in fruit plants, predominantly in Strawberry.

## Materials and Methods

### Identification of SBP-Box Genes in Rosacea

The genome data of four Rosaceae species were retrieved from their respective genome sequence websites: *F. vesca* from the Joint Genome Institute^1^ under accession no AEMH00000000; *Pyrus bretschneideri* from the GigaDB database^2^ under accession no. AJSU00000000; *Prunus persica* from the Phytozome database^3^ under the accession no AKXU00000000 and *Prunus mume* from the Genome Database for Rosaceae^4^ under accession no PRJNA171605. Two different methods were used to identify SBP-box genes in Rosacea species (*F. vesca, Pyrus bretschneideri, Prunus persica, Prunus mummer*, respectively). First we implemented BLASTP searches of the complete genome of these species using rice and *Arabidopsis* SBP-box protein sequences according to previous the method (Cao et al., 2017a,b) and second method was Hidden Markove Model (HMM) of SBP domain (PF03110) in these Rosacea species by using HMMER software with an *E*-value cut off of 0.001. Subsequently, we verified all sequences by using different tools Pfam, InterProScan database, NCBI and SMART database (Zdobnov and Apweiler, 2001; Bateman, 2002; Letunic et al., 2012; Finn et al., 2014).

### SBP-Box Genes Physical Localization and Gene Duplications

To identify chromosomal location of all SBP genes, genome annotation data was collected and mapped with MapInspect software. Gene duplication pattern of SBP genes in Strawberry (*F. vesca*), Chinese pear (*Pyrus bretschneideri*), Peach (*Prunus persica)*, Mei (*Prunus mume*) were analyzed by using MCScanX software and BLASTP (1*e*-10, identity >80%) (Wang et al., 2015).

### Phylogenetic Analysis and Motif Prediction and Gene Structure

The full-length sequence of SBP-box proteins from five plants were assembled to perform multiple sequence alignment by using CLUSTAL_X software (Thompson et al., 1997). After that we construct a phylogenetic tree using MEGA 5.1 software with NJ (neighbor-joining) method. During the phylogenetic analysis following parameters were set at bootstrap value 1000, Poisson correction and pairwise deletion. The SBP-box proteins conserved motif were obtained by MEME^5^ program (Bailey et al., 2015) with the following parameters a maximum number of motifs at 20 and motif width range 6–200 residues. Additionally, Pfam, InterProScan and SMART databases (Zdobnov and Apweiler, 2001; Bateman, 2002; Letunic et al., 2012) were used to annotate these motifs. All SBP-box genes DNA sequences and coding domain sequences (CDS) were used in Gene Structure Display Server tool^6^ to determine gene structure.

### Microsynteny Analysis and *cis*-Acting Element Analysis

In order to elaborate the precise region that containing SBP-box genes, Multiple Collinearity Scan toolkit (MCSscanX) was used to determine the microsynteny among these four species Strawberry (*F. vesca*), Chinese pear (*Pyrus bretschneideri*), Peach (*Prunus persica)*, and Mei (*Prunus mume*). SBP-box genes of *F. vesca, Pyrus bretschneideri, Prunus persica, Prunus mume* were ordered according to evolutionary tree classification. PlantCARE database was used to analyze putative *cis*-acting elements in the promoter region of SBP-box genes. Each gene upstream genomic sequence of 2000 bp with start codon (ATG) was used to determine *cis*-acting element.

### Calculation of Non-synonymous (*K*a) to Synonymous (*K*s) Substitutions

Synonymous and non-synonymous nucleotide substitutions rates were determined by using DnaSP v5.0 Software. *K*a/*K*s ratio of each duplicate gene pairs was determined to calculate the selection pressure and sliding window analysis was carried out to analyze the Synonymous (*K*s) and non-synonymous (*K*a) rates of all SBP-box genes encoding sites paralogs.

### Functional Divergence Analysis

Diverge 3.0 Beta 1 Software was used to examining functional divergence (I type II type) among each subfamily of SBP genes according to constructed phylogenetic tree. After gene duplication Type I and Type II functional divergence occurred, Type I functional divergence resulted to selectivity change in a specific amino acid, for example, change in the evolution rate. Type II functional divergence led to change the physical and chemical properties of amino acids. The MFE Theta coefficient 𝜃_1_ ranging from 0 to 1, indicating weak to strong functional divergence among gene classifications. The MFE Theta coefficient 𝜃_1_ of each SBP subfamily and Theta II (II type functional divergence) and posterior probability (QK) were calculated of each cluster (Gu, 1999; Gu et al., 2013).

### Expression Analysis of SBP Genes in Different Tissue of *Fragaria vesca*

RNA-seq data of gene expression of different tissue development was reported by Darwish et al. (2013) and retrieved from SGR GBrowse database. The gene expression was estimated if the FPKM value was equal or greater than to zero in minimum one of fourteen tissues. *F. vesca* SBP genes expression profile was visualized using R software (Bioconductor).

## Results

### Identification and Characteristics of SBP Family Genes in Rosaceae

**FIGURE 1:**
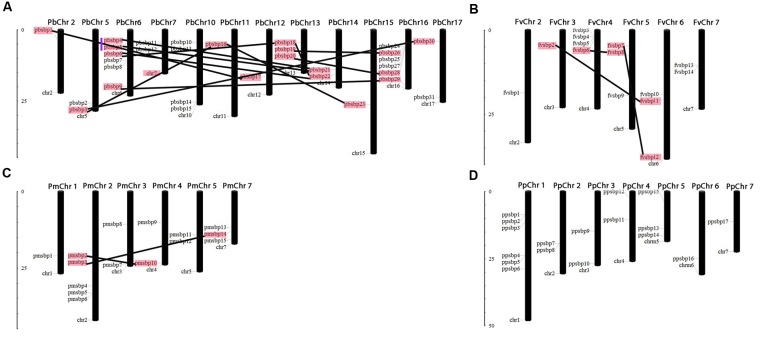
Chromosomal locations and region duplications of the SBP-box genes in the genome of Pear **(A)**, Strawberry **(B)**, Mei **(C)** and Peach **(D)**. The chromosome number is represented at the top of each chromosome and the left scale is in megabases (Mb). The segmental duplicated genes are represented by black lines and tandemly duplicated genes by blue vertical line.

For the identification of SBP transcription factor-coding genes of Strawberry (*F. vesca*), Chinese pear (*Pyrus bretschneideri*), Peach (*Prunus persica)*, and Mei (*Prunus mume*), BLASTP and HMM searches were performed against these entire genome sequences as well as for non-redundant protein databases. All identified sequences again verified for SBP domain through InterPro and SMART databases. As a result, we identified 14, 32, 17, and 17 SBP genes in *F. vesca*, *Pyrus bretschneideri, Prunus persica*, and *Prunus mume*, respectively (**Supplementary Table S2**). We designated all SBP genes according to their chromosomal positions from top to bottom (**Figure 1**). ExPASy server was used to calculate physicochemical parameters of SBP genes. The isoelectronic point or isoionic point (IP) is the pH at which the amino acid does not migrate in an electric field. The lowest IP value is 5.08 for *PbSBP10*, while the highest IP value is 9.64 for *PmSBP2* in these Rosaceae Species. The molecular weight ranges from 11522.23 to 127630.61 KDa for *PbSBP14* and *FvSBP5*. SBP genes, i.e., *PmSBP13* has the highest number of amino acids (1070) and *PbSBP14* has the lowest number of amino acids (100) while other physicochemical characteristics of the SBP genes were are also listed in **Supplementary Table S2**. In our results, SBP family genes in Strawberry less as compared to other studied species. We observed that there was no positive association between SBP family genes and genome size. For example, there was no significant difference between the genome size of Strawberry (240 Mb) and Pear (271.9 Mb) but the significant difference was found in SBP family genes. The number of SBP family genes of Peach (224.6 Mb) and Mei (231.04 Mb) had a parallel relationship with their genome size. Additionally, SBP family genes in Pear almost to be double as compared to Strawberry, Mei, and Peach. The number of chromosomes of Strawberry, Peach, and Mei are 14, 16, and 16, respectively, while the number of chromosomes of Pear is 34, suggesting that SBP family genes have gone through an expansion corresponding to chromosome variation.

### Phylogenetic Relationship and Gene Structure Analysis of SBP Family Genes in Rosacea

**FIGURE 2:**
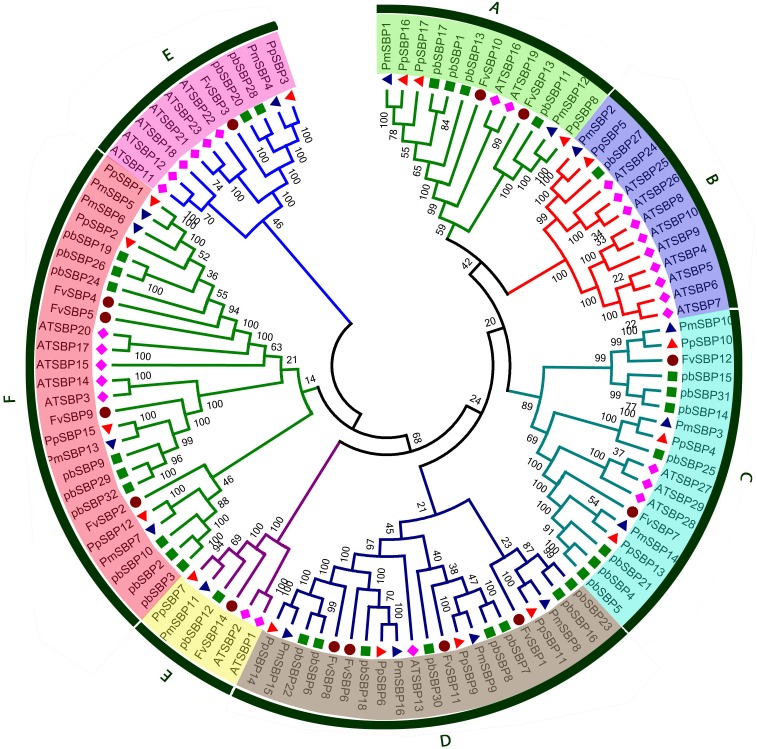
Phylogenetic tree of SBP protein from Strawberry, Pear, Peach, *Arabidopsis*, and Mei. The tree was generated with MEGA 5 software using the neighbor-joining method.

The phylogenetic relationship was analyzed among four Rosacea species and *Arabidopsis* of SBP family genes. Eighty SBP genes from four Rosacea species with thirty-two SBP genes from *Arabidopsis* were used to generating the evolutionary tree. Based on sequence similarity and topology, a phylogenetic tree was drawn with Mega 5.1 using neighbor-joining (NJ) method with bootstrapping 1000 times. As shown in **Figure 2**, 110 SBP genes could be categorized into seven groups (A–H). Each of the four studied species contributed minimum one member of SBP genes to each group, except the group B. Group F was composed the largest clade containing 26 SBP members that almost 23% of total SBP genes, while group E has the smallest clade containing only six members. In contrast, Rosacea species several SBP genes were not clustered with *Arabidopsis* SBP genes, these SBP genes may be developed in Rosacea species after diverging the last common ancestor or may be lost in *Arabidopsis*. In the evolutionary tree group, B and G contain more *Arabidopsis* SBP genes with few SBP genes from Rosacea species, suggesting that these genes in Rosacea may have been lost during the evolutionary process. The loss and birth of species-specific SBP genes caused functional divergence.

**FIGURE 3:**
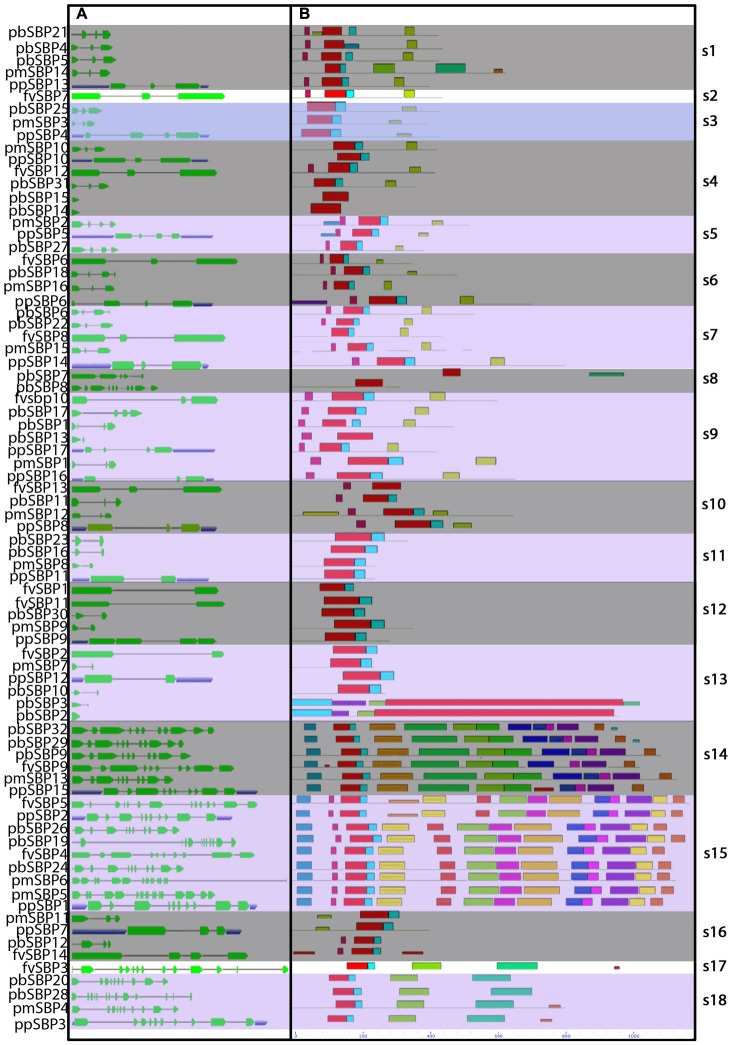
Gene structure and distribution of conserved motif of SBP-box genes in Rosaceae species. **(A)** Untranslated region (UTRs), introns, and exon indicated by the blue box, thin line, and green box. **(B)** Conserved motifs located on each gene with relative combined *P*-value.

Evolutionary studies indicated that structural diversity of genes is the main source to the evolution of gene family (Mercereau-Puijalon et al., 2002). To determine further insights into the structural diversity of SBP genes, we performed exon-intron structure analysis of each SBP gene (**Figure 3**). In general, most genes were clustered in the same group having a similar number of exon and intron, especially intron phases. As shown in **Figure 3**, group s12 and s13 comprise one intron and two exons, while the group s14 and s15 contain almost 11 to 13 introns. The splicing pattern of group s12, s13 showed the close relationship between these two groups but not for group s14 and s15. The result designated that acquire and loss of exon/intron events was occurred during the evolution of SBP family genes.

**FIGURE 4:**
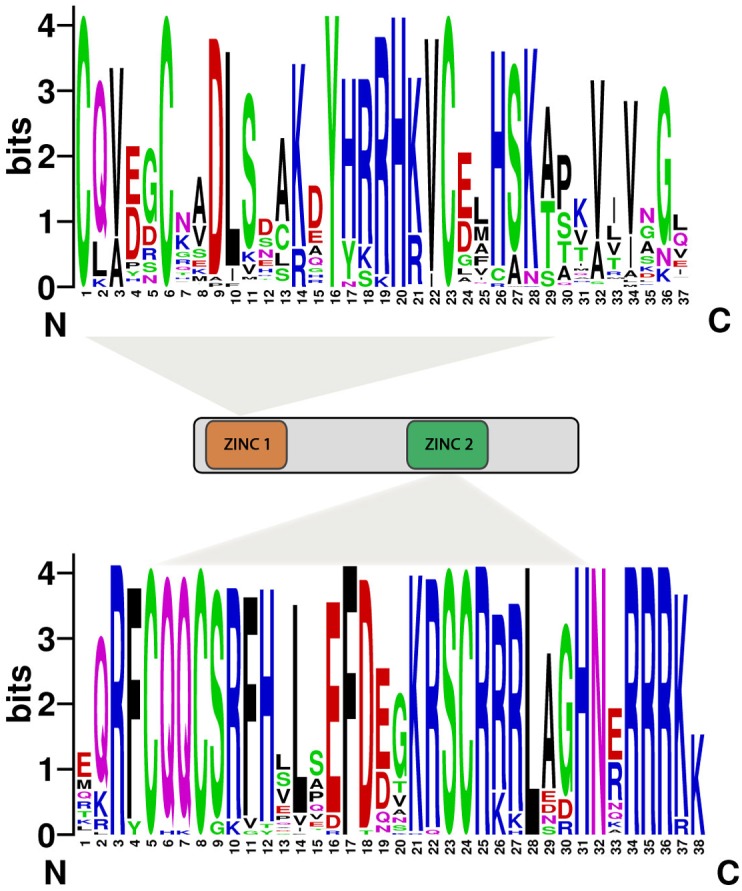
Domain compositions of SBP-box genes. Eighty SBP-box genes have both the characteristics of ZINC1 (Cys-Cys-His-Cys) and ZINC2 (Cys-Cys-Cys-His). The bit score represents the information content for each position in the sequence.

We searched conserved motif of 80 SBP proteins using MEME online web server. Subsequently, twenty motifs were identified in the SBP family genes (**Figure 3**). All SBP proteins contained Motif 1, showing have both Cys-Cys-Cys-His and Cys-Cys-His-Cys type Zinc finger (**Figure 4**) while the former was found less conserved than latter. Motif 9 and 15 was only present in group s14, s15, suggesting that proteins in these groups may be a share specific function. The majority within the same group exhibited similarity in motif compositions and intron-exon structure of SBP proteins supported the phylogenetic analysis of SBP family genes, while the difference among the different groups indicated their functions was diversified.

### Chromosomal Location and Duplication Events of SBP Family Genes in Rosacea

To determine the relationship between gene duplication and genetic divergence of SBP family genes among Rosacea species, we investigated chromosomal distribution in Strawberry, Pear, Peach, and Mei, respectively. All SBP genes distribution chromosomes map were drawn according to genome annotation, except one gene located on the scaffold. In pear, at chromosomes 2, 11, 12, and 15 only one SBP gene was found, respectively, 2 SBP genes were distributed on chromosomes 5, 7, 14, and 17; 3 genes on chromosomes 13; 4 genes on chromosome 10; 6 genes on chromosomes 16 and 6 (**Figure 1A**). In strawberry, only one SBP genes were located on chromosome 2 and 3, while the 4th chromosome contained a maximum number of four SBP genes and remaining chromosomes 5, 6, and 7 comprised 3, 3, and 2 genes, respectively (**Figure 1B**). In Mei, 1 SBP gene on chromosome 1; 2 SBP genes on chromosomes 3, 4, and 5; 3 SBP genes on chromosome 7 and maximum 5 SBP genes on chromosome 2 (**Figure 1C**). In peach, 1 SBP gene found on chromosomes 7 and 6; 2 SBP genes on chromosomes 2, 3, and 4; 3 SBP genes on chromosome 5 and maximum 6 SBP genes on chromosome 1 (**Figure 1D**). Expansion of any gene family mainly occurs through the tandem and segmental duplication events. In the present study, we identified segmental and tandem duplication events to elucidate the expansion of SBP genes in Rosacea species following their divergence (**Figure 1**). Genome-wide survey of gene duplications displayed 13, 3 and 2 pairs of duplication gene pairs in Pear, Strawberry, and Mei via segmental duplication and only one pair by tandem duplication in pear. We noticed that segmental duplication events in pear as compare to other studied species. Almost 30–45 million years ago the pear whole genome duplication event was occurred but not in Strawberry, Peach, and Mei, probably genome duplication is the main cause of SBP gene family expansion in pear (Wu et al., 2013).

**FIGURE 5:**
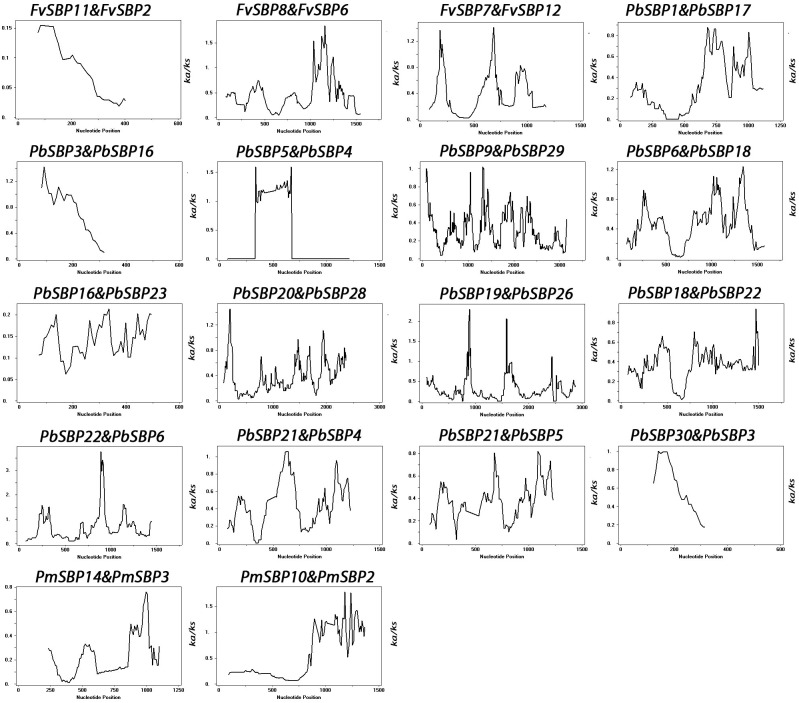
Sliding window plots of duplicated SBP-box genes in Rosaceae species. The window size is 150 bp, and the step size is 9 bp. The *X*-axis indicated the synonymous distance within each gene.

To determine driving force for gene duplication, we calculated non-synonymous (*K*a) and synonymous (*K*s) ratio for 18 duplicated gene pairs (**Supplementary Table S1**). Generally, *K*a/*K*s ratio of ≤1 was indicated as a useful cut-off value to determine genes under positive or neutral selection. In our study, all SBP gene pairs *K*a/*K*s ratio less than 1, suggesting that SBP genes are mainly under purifying selection. Furthermore, sliding window analysis was carried out to estimate the *K*a/*K*s ratios of the CDS sequence at different sites. This analysis showed some coding sites *K*a/*K*s ratios were more than one, recommending the positive selection in SBP genes (**Figure 5**).

### Microsynteny Analysis and *cis*-Element Analysis of SBP Family Genes

**FIGURE 6:**
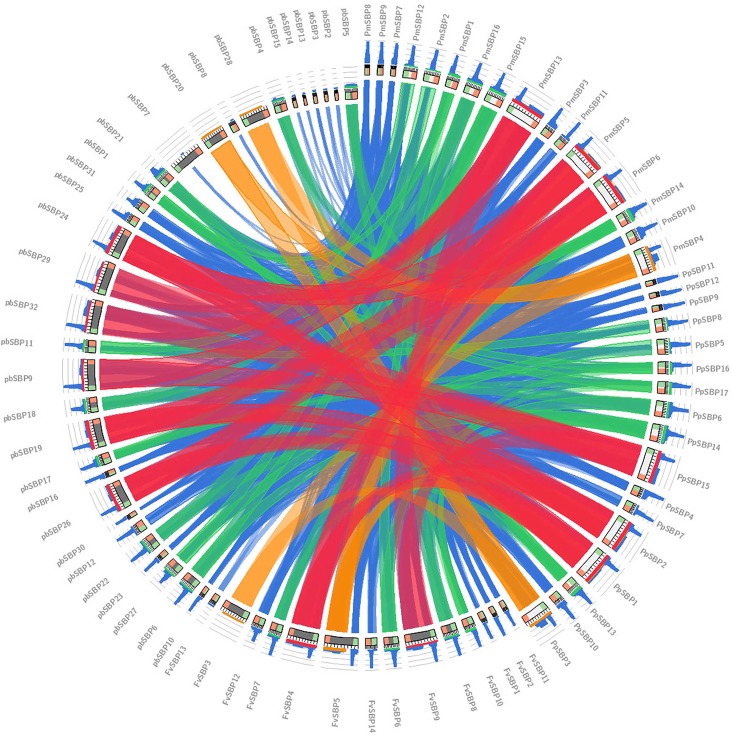
Eighty SBP-box genes collinearity analysis. Colored lines which connect two regions indicate collinearity regions between Strawberry, Pear, Mei, and Peach.

**FIGURE 7:**
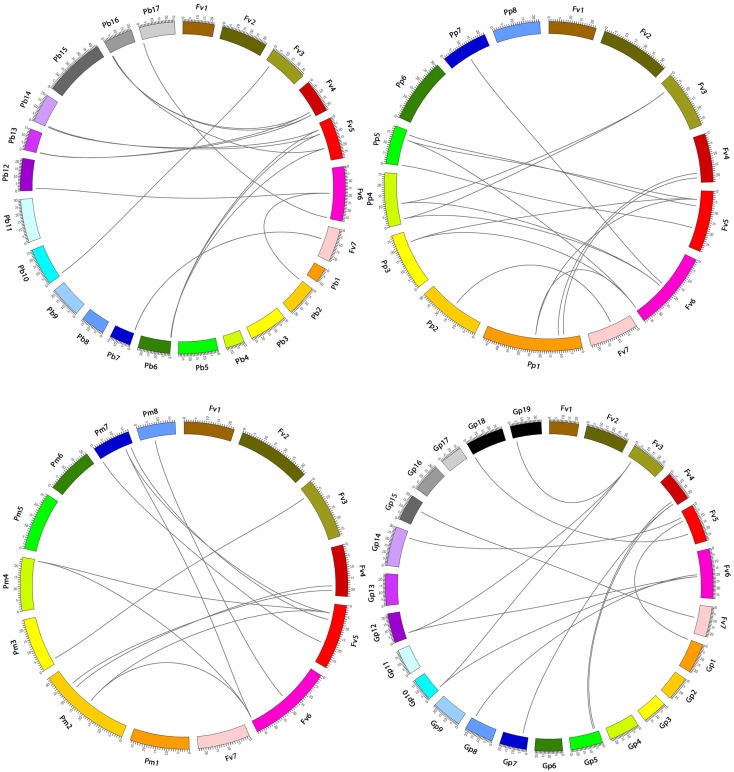
Microsynteny of regions among Strawberry, Pear, Mei, Peach, and Grape. The chromosomes number indicated by different colored boxes and labeled by Fv, Pb, Pm, Pp, and Gp. The different color box also represents the sequence lengths of chromosomes in megabases. The black line indicates the syntenic relationship among the SBP-box regions.

Interspecific microsynteny, using whole genome sequences can visualize the location of the homologous or orthologous genes (Cao et al., 2016b, 2017b). In our study, microsynteny analysis was performed across the four studied species Strawberry, Pear, Peach, and Mei, respectively (**Figure 6**). A total of 19, 18, 12, and 16 orthologous gene pairs were identified in the cross of the Strawberry and Peach, Strawberry and Pear, Strawberry and Mei, Strawberry and Grape. We identified some collinear gene pairs among strawberry and peach while could not identify strawberry and Mei, strawberry and Pear such as *FvSBP11/PpSBP9*, *FvSBP11/PpSBP11*, signifying that these orthologous gene pairs were developed after Mei diverged from the common ancestor of Peach and Pear. Similarly, some collinear genes recognized between strawberry/pear and strawberry/Mei while were not found in strawberry/peach. Subsequently, two or three SBP genes from Mei/Peach matched with one pair of Strawberry SBP gene (**Figure 7**). For example, *PpSBP11* and *PpSBP9* orthologous to *FvSBP11, PbSBP9* and *PbSBP29* to orthologous to *FvSBP9.*

For promoter analysis, eighty SBP genes 2000 bp upstream genomic DNA sequences with transcriptional start site (ATG). We observed a large amount of *cis*-acting element in the promoter region of SBP family genes (**Supplementary Table S3**). These results proposed that various type of *cis*-acting element in the same type SBP genes may have a different function. Most of the SBP genes contain regulatory elements AE-box, BOX-4, BOX-1, ABRE, ARE, ACE, MBS, and Skn-1. SBP genes contain AE-box, BOX-4, BOX-1 proposing that SBP genes involved in Light responsiveness. ABRE, HSE, and MBS were found in the promoter region implying that SBP genes are involved in drought, heat, and salt stress. Total 80 genes in which 65 genes have SKn-1 regulatory element indicating that SBP genes are involved in Endosperm expression. Similarly, 55 SBP genes contain circadian regulatory element showing that SBP genes may be involved in circadian control. These results showing that SBP duplicated genes may exhibit different regulatory features.

### Functional Divergence Analysis

The evolutionary study indicated that many plant species go through genome-wide or chromosomal duplication events under evolution process. The result of the duplication events, several genes denoted many related paralogs but different functions in the genome. Gene duplication provided the raw material for functional innovations, needed to scan amino acid sites responsible for functional divergence through sequence analysis of gene family (Gu et al., 2013). Functional divergence analysis was performed on all *SBP* genes amino acid sequence data by using Diverge 3.0 joined with a created Phylogenetic tree. The type I functional divergence directed to a change of functional limitation that was highly correlated with the evolution rate after gene duplication. Type II functional divergence led to change of physical and chemical properties of amino acid residues but can’t change in the functional limitation of the members after gene duplication. In order to identify, whether amino acid sites of SBP family genes responsible for functional divergence, we calculated Type I and Type II functional divergence based on the posterior analysis among subfamilies genes of SBP family.

We divided the SBP family genes into seven subfamilies followed by a phylogenetic tree. Type I functional divergence results showed that 𝜃_SE_ was not found among subfamily 3 vs. 6 and 4 vs. 5 (**Supplementary Table S4**). We calculated the posterior probability sites (QK > 0.90) that screen the key amino acid sites directing the functional divergence according to the previous research method (Cao et al., 2016a, 2017a). The significant type I functional divergence was determined in the 500th amino acid sites between group 2 vs. 6 and 3 vs. 7, in the 506th amino acid site between 4 vs. 6, and in the 500th site between 6 vs. 7. The group 2 vs. 6, 3 vs. 7, 4 vs. 6 and 6 vs. 7, *P*-values were less than 0.05, showing significant level. Type II functional divergence resulted changing the chemical and physical properties of an amino acid. In our results, we notice that Type II functional divergence was lesser or even negative between these groups. We detected group 1 vs. 2 had a 493th key amino acid site and the group 1 vs. 5 had a 530th key amino acid site for Type II functional divergence (**Supplementary Table S5**).

### Expression Profile of *F. vesca* SBP Genes

**FIGURE 8:**
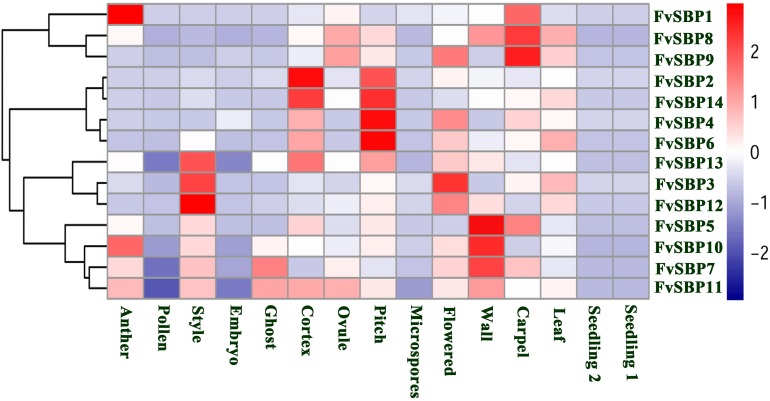
Expression profiling of Strawberry *FvSBP-box* genes fifteen samples from seedling, root, leaves, and flowering in different development stages. Blue and red colors indicate low-expression and high-expression, respectively.

A wide range of SBP family genes plays critical roles in different plant development process. In the lack of SBP gene mutants, the expression pattern may provide an indication to elucidate the important role of the different SBP genes in *F. vesca*. The expression level of *FvSBP* genes in fifteen tissues was shown by heat map representation (**Figure 8**). To explore the expression diversity of SBP family genes in *F. vesca*, we analyzed RNA-seq transcriptomic dataset obtained from SGR GBrowse database to investigate the expression pattern of each SBP gene during different development process (anther, Pollen, embryo, ghost, cortex, ovule, seedling, pollen, style, pith, wall, and leaf). The SBP family genes of *F. vesca* were classified into four groups on the basis of their hierarchical clustering on their expression patterns. All the SBP genes showed their divergent expression level among all the development processes except seedling, indicating that SBP genes were active and very important during the development processes (**Figure 8**). Subsequently, most of the SBP genes expression displayed developmental stage specificity, for example, *FvSBP2, FvSBP14*, *FvSBP4* and *FvSBP6* in the Pitch and *FvSBP13*, *FvSBP3*, and *FvSBP12* in Style and *FvSBP5*, *FvSBP10*, and *FvSBP17* in Wall. Fourteen *FvSBP* genes expressed more abundantly in flowering-related tissues as compared to other tissues, specifically *FvSBP8, FvSBP14, FvSBP13, FvSBP10*, and *FvSBP5*, which recommended that *SBP* genes necessity in the Flowering process. The expression profiles during seedling development stage have no more difference with leaf development stage but seem big difference with flowers. We observed that *FvSBP13*, *FvSBP6*, and *FvSBP4* were expressed among all the tissue, revealing that these genes are most important and persistent during the development process of *F. vesca*.

## Discussion

SQUAMOSA promoter-binding-protein (SBP-box) gene family encodes SBP proteins which play a vital role in plant development, signaling and defense mechanisms. In the present study, we identified and analyzed 80 SBP family genes in four Rosacea species and investigated their expression profile in Strawberry during developmental stages. Many plants whole genome sequence available that offer a useful tool for genome-wide analysis of SBP family genes.

Although SBP gene family in some plants, such as *Arabidopsis*, Moso Bamboo, Pepper, Chinese cabbage, and rice have been studied while comprehensive molecular evolutionary study remains elusive. In the present study, the first time we comprehensively and systematically analyzed the SBP family genes in Four Rosacea species, including gene structure, phylogenetic analysis, promoter region, chromosomal location, duplication events and microsyneteny and expression profile. The functions of most SBP family genes are unknown, while a basic function can be illustrated by the evolution of the SBP family genes in higher plants. The SBP family genes are plant-specific transcription factor family with no homologs in human, animal, and bacteria (Cardon et al., 1997; Pan et al., 2017).

SBP gene family in four studied Rosacea species was shown along with *Arabidopsis thaliana* similarity and conservation. SBP gene family could be distributed into seven groups on the basis of structural similarities. Zhang et al. (2016) reported that SBP gene family could be divided into six groups of pepper on the basis of phylogenetic analysis. The structural organization of intron-exon showed high variation ranges from 1 to 11 in one gene, similar to *Arabidopsis*. A maximum number of introns reported in pepper ranges from 0 to 11 (Zhang et al., 2016) and in moso bomboo ranges from 0 to 10 (Pan et al., 2017). The resemblance and structural variation among these motifs can be further studied to provide novel information.

Gene duplication events (segmental and tandem) are the major driving forces for breeding novel genes and gene family expansion, which can support the organisms to adapt different complex environments. We identified segmental and tandem duplications of the SBP genes in four studied Rosacea species. A total of 3, 13, and 2 duplication events were recognized in Strawberry, Pear, and Mei, most of them segmental duplications and only one tandem duplication *PbSBP4/PbSBP5* was noticed. A large number of duplication events were observed in Pear as compare to Strawberry and Mei, and no duplication event were identified in Peach. Four to five billion years ago the complete genome duplication/triplication and genome replication occurred in Pear (Velasco et al., 2010; Cao et al., 2017a) that amplified the chromosome number from 9 to 17, might be due to this a large number of SBP family genes duplication events occur in Pear. Additionally, one *PbSBP* could not map on any chromosome, may be due to quality or heterozygosity sequence of Pear genome. The SBP family genes have a large number of responsive hormone-related *cis*-acting elements in the upstream regulatory sequence. Among them, 41 SBP contains drought/salt stress response elements (ABRE), 29 SBP genes with light responsive elements (ACE), 28 SBP genes containing light responsive elements (AE-box), 50 SBP genes containing Methyl jasmonate (MeJA) response element (CGTCA), 48 SBP genes have salicylic acid response element (TCA). These following hormones are commonly involved in the signal pathways of mature senescence or stress response, suggesting that SBP family genes participate in fruit ripening and stress. In addition, we identified various light responsive elements in the regulatory upstream sequence suggesting that the expression of SBP family genes was regulated by light.

Pan et al. (2017) expose that most SBP-BOX genes highly expressed in all developmental stages, while *PeSPL7* the ortholog of *OsSPL14* showed high expression level in a panicle. Our finding suggested that SBP genes are expressed at high levels and widely in the different organs or developmental stages while it is possible to suggest that SBP family genes have no specific role in the development of leaf, seedling, and ovule. Zhang et al. (2016) observed only one SBP gene *caSBP02* expression in root and leaf that also supported our results. The *K*a/*K*s of 18 duplicate gene pairs suggesting that purifying selection might be largely responsible for retaining the function of SBP proteins from the four Rosacea species (Strawberry, Pear, Peach, and Mei). In addition, the *K*a/*K*s of duplicated gene pairs less than 0.5 except only one duplicate gene pair, suggesting that these genes go through slow evolutionary non-diversification resulting duplication. Furthermore, we noticed that strong positive selection in the coding region of SBP gene pairs showing functional differentiation. DIVERGE software was used for sequence analysis of seven subfamily genes of SBP-BOX. The analysis of type I and type II functional divergence identify the important amino acid sites that may lead to functional differentiation of SBP genes, our study offers new insights for future researchers discovering SBP family genes functional divergence.

## Conclusion

We have performed genome-wide analysis of SBP-BOX transcriptional factors in Four Rosacea species (Strawberry, Pear, Peach, and Mei). In this study, we used systematic analysis including phylogenetic relationship, chromosomal location, gene structure, gene duplication, conserved motifs, conserved microsyneteny, promoter region, expression profile, sliding window, and functional divergence, which may be related to their biological function was carried out. A total of 80 non-redundant SBP family genes were identified and divided into seven groups according to phylogenetic analysis. Remarkably, microsyneteny analysis of SBP-BOX family in Rosacea species indicated that genome duplication contributed to the expansion of SBP-BOX genes in Rosacea. The expression pattern of SBP-BOX genes in various tissues suggested that SBP genes may function in growth and organ development. Identification and analysis of these SBP family genes in Rosacea species provide a basic understanding and foundation for extrapolation of SBP-BOX genes function for future studies in Rosacea.

## Author Contributions

MA and YuC conceived and designed the experiments. MA, YuC, XC, AS, XS, JG, and YoC contributed reagents/materials/analysis tools. YoC and JG provided guidance on the whole manuscript. MA wrote the article.

## Conflict of Interest Statement

The authors declare that the research was conducted in the absence of any commercial or financial relationships that could be construed as a potential conflict of interest. The reviewers WZ and SM and handling Editor declared their shared affiliation.

## Footnotes

1 http://www.jgi.doe.gov/

2 http://gigadb.org/site/index

3 https://phytozome.jgi.doe.gov/pz/portal.html

4 http://www.rosaceae.org/

5 http://meme.sdsc.edu/meme/

6 http://gsds.cbi.pku.edu.cn/

## Supplementary Material

TABLE S1 Synonymous and non-synonymous substitution rates for the duplication events in Rosaceae species.

TABLE S2 The detailed information of *SBP-box* genes from Strawberry, Pear, Peach, and Mei.

TABLE S3 *cis*-Acting element of 80 SBP protein sequence.

TABLE S4 Analysis of type I functional divergence.

TABLE S5 Analysis of type II functional divergence.
