# Supplementary material for: Genome-Wide Analysis Characterization and Evolution of SBP Genes in Fragaria vesca, Pyrus bretschneideri, Prunus persica and Prunus mume
Source: Front Genet. 2018 Mar 2;9:64. doi: 10.3389/fgene.2018.00064 (PMC5841269; doi:10.3389/fgene.2018.00064)
Supplement: TABLE S1 — Synonymous and non-synonymous substitution rates for the duplication events in Rosaceae species. [file Table_1.DOCX]

| Duplicated gene 1 | Duplicated gene 2 | Ks | ka | Ka/ks | selection | Duplication Type |
| --- | --- | --- | --- | --- | --- | --- |
| FvSBP6 | FvSBP8 | 1.6142 | 0.6508 | 0.403172 | Purifying | Segmental |
| FvSBP7 | FvSBP12 | 2.7032 | 0.7308 | 0.270346 | Purifying | Segmental |
| FvSBP11 | FvSBP2 | 0.2075 | 0.0643 | 0.309879 | Purifying | Segmental |
| FvSBP1 | FvSBP17 | 0.1363 | 0.0356 | 0.261189 | Purifying | Segmental |
| PbSBP3 | PbSBP16 | 1.1566 | 0.5639 | 0.48755 | Purifying | Segmental |
| PbSBP5 | PbSBP4 | 1.5645 | 0.7752 | 0.495494 | Purifying | Tandem |
| PbSBP6 | PbSBP18 | 1.6192 | 0.6674 | 0.412179 | Purifying | Segmental |
| PbSBP9 | PbSBP29 | 0.1667 | 0.0491 | 0.294541 | Purifying | Segmental |
| PbSBP16 | PbSBP23 | 0.2393 | 0.0339 | 0.141663 | Purifying | Segmental |
| PbSBP18 | PbSBP22 | 2.0103 | 0.7216 | 0.358951 | Purifying | Segmental |
| PbSBP19 | PbSBP26 | 0.2047 | 0.0544 | 0.265755 | Purifying | Segmental |
| PbSBP20 | PbSBP28 | 0.211 | 0.0731 | 0.346445 | Purifying | Segmental |
| PbSBP21 | PbSBP4 | 0.2514 | 0.1133 | 0.450676 | Purifying | Segmental |
| PbSBP21 | PbSBP5 | 1.4633 | 1.3185 | 0.901046 | Purifying | Segmental |
| PbSBP22 | PbSBP6 | 0.122 | 0.0623 | 0.510656 | Purifying | Segmental |
| PbSBP30 | PbSBP3 | 1.4387 | 0.5936 | 0.412595 | Purifying | Segmental |
| PmSBP10 | PmSBP2 | 1.1002 | 1.0117 | 0.91956 | Purifying | Segmental |
| PmSBP14 | PmSBP3 | 1.7448 | 0.395 | 0.226387 | Purifying | Segmental |
