# Supplementary material for: Genome-Wide Analysis Characterization and Evolution of SBP Genes in Fragaria vesca, Pyrus bretschneideri, Prunus persica and Prunus mume
Source: Front Genet. 2018 Mar 2;9:64. doi: 10.3389/fgene.2018.00064 (PMC5841269; doi:10.3389/fgene.2018.00064)
Supplement: TABLE S2 — The detailed information of SBP-box genes from Strawberry, Pear, Peach, and Mei. [file Table_2.DOCX]

| **Gene Name** | **Gene Identifier** | **Chromosome** | **5′ End** | **3′ End** | **Length** | **Molecular weight** | **theoretical PI** | **Instability index** | **Aliphatic index** |
| --- | --- | --- | --- | --- | --- | --- | --- | --- | --- |
| FvSBP11 | mrna17738 | chr6 | 20658494 | 20659706 | 178 | 20454 | 9.44 | 74.95 | 49.27 |
| FvSBP2 | mrna29726 | chr3 | 4429085 | 4430399 | 157 | 17887.66 | 7.65 | 100.35 | -1.257 |
| FvSBP1 | mrna00196 | chr2 | 18617378 | 1861864 | 187 | 20738.9 | 9.02 | 62.78 | -1.133 |
| FvSBP10 | mrna22660 | chr6 | 19024604 | 19028037 | 380 | 41035.16 | 9.47 | 57.52 | -0.795 |
| FvSBP8 | mrna26117 | chr5 | 7898148 | 7900667 | 514 | 56456.58 | 8.5 | 54.93 | -0.683 |
| FvSBP9 | mrna29149 | chr5 | 19464134 | 19468831 | 1071 | 118588.51 | 8.56 | 61.14 | -0.516 |
| FvSBP6 | mrna04565 | chr4 | 26518038 | 26520978 | 500 | 55986.19 | 6.74 | 52.27 | -0.67 |
| FvSBP14 | mrna04786 | chr7 | 10489209 | 10491413 | 403 | 45207.56 | 9.62 | 66.51 | -0.781 |
| FvSBP5 | mrna22889 | chr4 | 20846585 | 20854585 | 1166 | 127630.61 | 6.29 | 49.12 | -0.317 |
| FvSBP4 | mrna22888 | chr4 | 20839800 | 20845275 | 1033 | 114676.16 | 6.46 | 46.43 | -0.38 |
| FvSBP7 | mrna06295 | chr5 | 4600569 | 4602762 | 411 | 45504.99 | 9 | 58.54 | -0.874 |
| FvSBP12 | mrna01338 | chr6 | 37724191 | 37726390 | 380 | 42622.85 | 9.06 | 47.09 | -0.569 |
| FvSBP3 | mrna06692 | chr4 | 18486247 | 18495919 | 937 | 104196.81 | 7.78 | 60.93 | -0.386 |
| FvSBP13 | mrna04784 | chr7 | 10463184 | 10463861 | 225 | 24409.36 | 7.77 | 41.7 | -0.471 |
| PpSBP11 | ppa022739m | chr4 | 10508967 | 10510314 | 162 | 18482.6 | 9.14 | 83.07 | -1.177 |
| PpSBP12 | ppa012607m | chr4 | 2410899 | 2412783 | 162 | 18620.05 | 5.61 | 101.39 | -1.519 |
| PpSBP9 | ppa011968m | chr3 | 4747483 | 14749256 | 189 | 21523.1 | 9.26 | 61.8 | -1.17 |
| PpSBP8 | ppa024285m | chr2 | 19267582 | 19271212 | 419 | 46180.72 | 8.65 | 64.14 | -0.765 |
| PpSBP5 | ppa005013m | chr1 | 23865042 | 23870556 | 481 | 52544.1 | 8.49 | 42.89 | -0.74 |
| PpSBP16 | ppa007056m | chr6 | 24912779 | 24917334 | 384 | 41338.83 | 9.47 | 54.14 | -0.678 |
| PpSBP17 | ppa021582m | chr7 | 11146101 | 11151079 | 383 | 41505.97 | 9.59 | 51.07 | -0.756 |
| PpSBP6 | ppa017695m | chr1 | 28899733 | 28904841 | 499 | 55061.43 | 6.74 | 53.49 | -0.563 |
| PpSBP14 | ppa003644m | chr5 | 16610659 | 16614654 | 559 | 61263.08 | 7.07 | 46.64 | -0.573 |
| PpSBP15 | ppa000682m | chr5 | 377713 | 384289 | 1037 | 114733.48 | 8.44 | 56.94 | -0.501 |
| PpSBP4 | ppa023657m | chr1 | 23739022 | 23742227 | 300 | 32852.81 | 9.12 | 66.02 | -0.576 |
| PpSBP7 | ppa008560m | chr2 | 19258152 | 19260995 | 327 | 36255.86 | 8.95 | 64.73 | -0.803 |
| PpSBP2 | ppa000792m | chr1 | 8549838 | 8556894 | 1002 | 111298.84 | 6.34 | 49.28 | -0.36 |
| PpSBP1 | ppa000690m | chr1 | 8542094 | 8549335 | 1035 | 114891.4 | 7 | 50.24 | -0.398 |
| PpSBP13 | ppa006611m | chr5 | 13587845 | 13591533 | 403 | 44188.1 | 8.27 | 52.54 | -0.682 |
| PpSBP10 | ppa007202m | chr3 | 26849613 | 26852080 | 320 | 35617.1 | 8.96 | 61.37 | -0.658 |
| PpSBP3 | ppa001613m | chr1 | 11351929 | 11359238 | 850 | 95352.29 | 5.99 | 49.82 | -0.397 |
| PbSBP1 | Pbr019232.1 | Chr2 | 3604773 | 3608047 | 394 | 42355.01 | 9.56 | 48.64 | -0.618 |
| PbSBP3 | Pbr000386.1 | Chr5 | 25705287 | 25705821 | 126 | 14058.49 | 4.94 | 86.63 | -0.949 |
| PbSBP2 | Pbr000388.1 | Chr5 | 25692936 | 25693289 | 117 | 12930.38 | 5.42 | 81.52 | -0.932 |
| PbSBP5 | Pbr017710.1 | Chr6 | 419412 | 421715 | 424 | undefined | undefined | 48.62 | -0.624 |
| PbSBP4 | Pbr037448.1 | Chr6 | 36730 | 39025 | 428 | undefined | undefined | 43.07 | -0.603 |
| PbSBP8 | Pbr035125.1 | Chr6 | 10965770 | 10966607 | 233 | 25975.07 | 6.37 | 43.8 | -0.797 |
| PbSBP7 | Pbr035121.1 | Chr6 | 10940046 | 10944532 | 1118 | 124783.68 | 5.52 | 42.24 | -0.491 |
| PbSBP9 | Pbr015125.1 | Chr6 | 19847252 | 19851986 | 1074 | 118895 | 8.12 | 54.67 | -0.485 |
| PbSBP6 | Pbr020183.1 | Chr6 | 4449472 | 4452290 | 541 | 59199.99 | 8.12 | 53.63 | -0.549 |
| PbSBP11 | Pbr015983.1 | Chr7 | 4563724 | 4567820 | 419 | 46765.72 | 8.32 | 58.97 | -0.746 |
| PbSBP12 | Pbr015985.1 | Chr7 | 4600263 | 4602657 | 346 | 38474.34 | 8.63 | 58.41 | -0.84 |
| PbSBP13 | Pbr003814.1 | Chr10 | 8047192 | 8047675 | 142 | 14751.41 | 9.17 | 42.34 | -0.47 |
| PbSBP14 | Pbr002300.1 | Chr10 | 25328395 | 25328697 | 100 | 11522.23 | 9.47 | 48.69 | -0.791 |
| PbSBP15 | Pbr002303.1 | Chr10 | 25348096 | 25348482 | 128 | 14673.71 | 8.78 | 52.07 | -0.865 |
| PbSBP10 | Pbr016235.1 | Chr10 | 4285044 | 4286828 | 174 | 19795.24 | 5.08 | 109.39 | -1.371 |
| PbSBP16 | Pbr038289.1 | Chr11 | 5048241 | 5050844 | 189 | 21218.33 | 9.33 | 71.68 | -1.277 |
| PbSBP17 | Pbr035851.1 | Chr12 | 16403113 | 16405468 | 377 | 40373.64 | 9.34 | 54.2 | -0.687 |
| PbSBP20 | Pbr014789.1 | Chr13 | 11846721 | 11852235 | 799 | 89772.48 | 7.02 | 51.38 | -0.375 |
| PbSBP19 | Pbr018552.1 | Chr13 | 7547800 | 7562553 | 983 | 109108.22 | 6.28 | 50.3 | -0.396 |
| PbSBP18 | Pbr029987.1 | Chr13 | 4410955 | 4414560 | 517 | 57650.11 | 7.86 | 53.11 | -0.621 |
| PbSBP22 | Pbr007182.1 | Chr14 | 15105074 | 15107518 | 499 | 54602.02 | 8.76 | 53 | -0.564 |
| PbSBP21 | Pbr036874.1 | Chr14 | 14032938 | 14036120 | 432 | undefined | undefined | 54.19 | -0.706 |
| PbSBP23 | Pbr017399.1 | Chr15 | 26114207 | 26116185 | 189 | 21029.15 | 9.1 | 64.78 | -1.215 |
| PbSBP29 | Pbr001211.1 | Chr16 | 19879502 | 19884380 | 1135 | 125890.33 | 8.65 | 59.78 | -0.469 |
| PbSBP24 | Pbr037762.1 | Chr16 | 5666240 | 5672672 | 1002 | 110535.56 | 6.93 | 44.64 | -0.348 |
| PbSBP25 | Pbr015152.1 | Chr16 | 5914728 | 5915878 | 311 | 35195.17 | 9.52 | 54.65 | -0.896 |
| PbSBP28 | Pbr011184.3 | Chr16 | 6660690 | 6670980 | 936 | 105050.47 | 6.58 | 53.51 | -0.386 |
| PbSBP27 | Pbr015161.1 | Chr16 | 6025512 | 6027615 | 469 | 51392.52 | 6.81 | 46.35 | -0.749 |
| PbSBP26 | Pbr037761.1 | Chr16 | 5674841 | 5679892 | 972 | 107915.65 | 6.07 | 55.69 | -0.442 |
| PbSBP30 | Pbr026854.1 | Chr17 | 4007456 | 4008846 | 163 | 18635.91 | 9.64 | 71.45 | -1.251 |
| PbSBP31 | Pbr038099.1 | Chr17 | 23668362 | 23670279 | 334 | 37558.38 | 8.92 | 49.61 | -0.723 |
| PbSBP32 | Pbr041959.1 | scaffold964.0 | 79964 | 84851 | 1153 | undefined | undefined | 59.35 | -0.451 |
| PmSBP8 | Pm011381 | chr3 | 10704691 | 10705520 | 162 | 18454.55 | 9.12 | 80.43 | -1.201 |
| PmSBP9 | Pm014154 | chr4 | 10035344 | 10036476 | 189 | 21654.16 | 9.16 | 62.28 | -1.237 |
| PmSBP7 | Pm010075 | chr3 | 2404865 | 240623 | 161 | 18591.17 | 5.76 | 100.28 | -1.509 |
| PmSBP12 | Pm017778 | chr5 | 14048386 | 0.999994 | 425 | 46941.75 | 8.61 | 62.9 | -0.724 |
| PmSBP1 | Pm002693 | chr1 | 20969892 | 20973764 | 383 | 41111.56 | 9.47 | 52.24 | -0.67 |
| PmSBP16 | Pm030597 | scaffold56 | 960704 | 963861 | 488 | 54047.27 | 7.05 | 51.85 | -0.599 |
| PmSBP15 | Pm025028 | chr7 | 15633103 | 15635711 | 551 | 60378.15 | 6.89 | 43.28 | -0.551 |
| PmSBP13 | Pm022881 | chr7 | 116896 | 121543 | 1070 | 118402.72 | 8.42 | 55.32 | -0.482 |
| PmSBP11 | Pm017777 | chr5 | 14035545 | 14037080 | 301 | undefined | undefined | 66.75 | -0.832 |
| PmSBP14 | Pm024528 | chr7 | 12867067 | 12869465 | 403 | 44262.18 | 8.45 | 53.33 | -0.694 |
| PmSBP10 | Pm016138 | chr4 | 23333197 | 23334845 | 389 | 43171.72 | 8.42 | 55.61 | -0.636 |
| PmSBP2 | Pm007020 | chr2 | 20851318 | 20853843 | 480 | 52697.36 | 8.48 | 44.39 | -0.767 |
| PmSBP3 | Pm007035 | chr2 | 20960217 | 20961741 | 316 | 34709.11 | 9.64 | 57.12 | -0.606 |
| PmSBP4 | Pm008092 | chr2 | 30819676 | 30826337 | 816 | 91882.59 | 6.3 | 51.05 | -0.408 |
| PmSBP5 | Pm008475 | chr2 | 33461309 | 33467634 | 1034 | 114965.71 | 6.85 | 49.3 | -0.396 |
| PmSBP6 | Pm008474 | chr2 | 33463398 | 33463548 | 1027 | 114398.81 | 6.73 | 49.47 | -0.39 |

The detailed information of *SBP-box* genes from Strawberry, Pear, Peach and Mei.
