# Supplementary material for: Genome-Wide Analysis Characterization and Evolution of SBP Genes in Fragaria vesca, Pyrus bretschneideri, Prunus persica and Prunus mume
Source: Front Genet. 2018 Mar 2;9:64. doi: 10.3389/fgene.2018.00064 (PMC5841269; doi:10.3389/fgene.2018.00064)
Supplement: TABLE S4 — Analysis of type I functional divergence. [file Table_4.docx]

| SBP-box Subfamilies | ᶿ_1_ | ᶿSE | ᶿLRT | Q_K_>0.9 | P |
| --- | --- | --- | --- | --- | --- |
| I vs II | 0.33497 | 0.233538 | 2.694733 |  |  |
| I VS III | 0.232837 | 0.210191 | 1.068235 |  |  |
| I VS 4 | 0.049976 | 0.171234 | 3.172762 |  |  |
| I VS 5 | 0.154957 | 0.206633 | 2.330528 |  |  |
| I VS 6 | 0.399362 | 0.216271 | 0.369392 |  |  |
| I VS 7 | 0.31825 | 0.315105 | 0.368467 |  |  |
| 2 VS 3 | 0.539604 | 0.256427 | 1.017706 |  |  |
| 2VS 4 | 0.448132 | 0.239217 | 4.270156 |  |  |
| 2 VS 5 | 0.329391 | 0.243294 | 0.855791 |  |  |
| 2 VS 6 | 0.928199 | 0.260928 | 3.004797 | 0.969534 | P<0.5 |
| 2 VS 7 | 0.741047 | 0.357605 | 2.642549 |  |  |
| 3 VS 4 | 0.403595 | 0.224941 | 4.648873 |  |  |
| 3 VS 5 | 0.481329 | 0.248699 | 1.966398 |  |  |
| 3 VS 6 | 0.0031 | 0.159366 | 0 |  |  |
| 3 VS 7 | 0.925585 | 0.3499 | 7.097874 | 0.999282 | P<0.5 |
| 4 VS 5 | -0.13683 | 0.147366 | 0 |  |  |
| 4 VS 6 | 0.699557 | 0.236463 | 2.247886 | 0.929449 | P<0.5 |
| 4 VS 7 | -0.09091 | 0.256188 | 0.231273 |  |  |
| 5 VS 6 | 0.679783 | 0.24953 | 0.018716 |  |  |
| 5 VS 7 | -0.4495 | 0.21915 | 1.097206 |  |  |
| 6 VS 7 | 0.702698 | 0.326886 | 3.449696 | 0.999151 | P<0.5 |

**Table .4 Analysis of type I functional divergence.**
