# Supplementary material for: Genome-Wide Analysis Characterization and Evolution of SBP Genes in Fragaria vesca, Pyrus bretschneideri, Prunus persica and Prunus mume
Source: Front Genet. 2018 Mar 2;9:64. doi: 10.3389/fgene.2018.00064 (PMC5841269; doi:10.3389/fgene.2018.00064)
Supplement: TABLE S5 — Analysis of type II functional divergence. [file Table_5.docx]

| SBP-box Subfamilies | ᶿ_11_ | ᶿSE | QK>0.9 | P<0.5 |
| --- | --- | --- | --- | --- |
| I vs II | 0.062677 | 0.207922 | 0.93437 | P<0.5 |
| I VS III | 0.063292 | 0.219079 |  |  |
| I VS 4 | 0.239236 | 0.199382 |  |  |
| I VS 5 | 0.152898 | 0.236246 | 0.998992 | P<0.5 |
| I VS 6 | 0.158766 | 0.16094 |  |  |
| I VS 7 | 0.417712 | 0.152607 |  |  |
| 2 VS 3 | -0.120188 | 0.224814 |  |  |
| 2VS 4 | -0.086751 | 0.229107 |  |  |
| 2 VS 5 | -0.171034 | 0.259765 |  |  |
| 2 VS 6 | 0.000151 | 0.166642 |  |  |
| 2 VS 7 | 0.161843 | 0.185924 |  |  |
| 3 VS 4 | -0.019376 | 0.226123 |  |  |
| 3 VS 5 | -0.171688 | 0.25991 |  |  |
| 3 VS 6 | 0.102281 | 0.156547 |  |  |
| 3 VS 7 | 0.3217 | 0.16768 |  |  |
| 4 VS 5 | -0.204903 | 0.254016 |  |  |
| 4 VS 6 | 0.047739 | 0.166058 |  |  |
| 4 VS 7 | 0.094671 | 0.19086 |  |  |
| 5 VS 6 | -0.157163 | 0.211268 |  |  |
| 5 VS 7 | 0.28536 | 0.176664 |  |  |
| 6 VS 7 | 0.457091 | 0.094674 |  |  |

**Table.5 Analysis of type II functional divergence.**
